# Combined inhibition of STAT and Notch signalling effectively suppresses tumourigenesis by inducing apoptosis and inhibiting proliferation, migration and invasion in glioblastoma cells

**DOI:** 10.1080/19768354.2021.1942983

**Published:** 2021-06-25

**Authors:** Israt Jahan Tulip, Sung-Ok Kim, Eun-Jung Kim, Jaebong Kim, Jae Yong Lee, Hyunggee Kim, Sung-Chan Kim

**Affiliations:** a Department of Biochemistry, Institute of Cell Differentiation and Aging, College of Medicine, Hallym University, Chuncheon, Republic of Korea; b Department of Biotechnology, School of Life Sciences and Biotechnology, Korea University, Seoul, Republic of Korea

**Keywords:** Glioblastoma, STAT signalling, Jagged, Notch signalling

## Abstract

Glioblastoma multiforme (GBM) is the most aggressive primary brain cancer and this is due to cancer cells being apoptosis-resistant and having increased cell proliferation, migration, invasion, and angiogenesis properties. Previous studies have indicated both STAT and Notch pathways being important for initiation and progression in GBM. In this work, we first studied the effects of STAT inhibitors on Notch signalling using small molecule STAT inhibitors. It was observed that STAT inhibitors surprisingly activated Notch signalling by inducing NICD and Notch target genes in GBM cells. Thus, we aimed to combine STAT inhibitor treatment with a Notch pathway inhibitor and study effects on GBM tumourigenesis. STAT5 inhibitor (Pimozide) and STAT3 inhibitor (S3I-201) were individually used in combination with γ-secretase inhibitor (DAPT), an inhibitor of Notch signalling, in a panel of GBM cells for cell proliferation and epithelial plasticity changes. Compared with single-agent treatments, combinatorial treatments with the STAT and Notch inhibitors significantly increased apoptosis in the treated cells, impairing cell proliferation, migration, and invasion. These findings suggest that concurrent blocking of STAT and Notch signalling pathways could provide added therapeutic benefit for the treatment of glioblastoma.

## Introduction

Gliomas are tumours of glial cell origin in the brain and the spinal cord. Glioblastoma multiforme (GBM), as the WHO grade IV astrocytoma, is the most aggressive primary brain tumour derived from gliomas and it has high morbidity and poor prognosis. GBM comprises 12–15% of all intracranial tumours and 50–60% of astrocytic tumours (Zhang et al. 2012). GBM is also of two types with primary GBM which arises de novo and secondary GBM which develops from a lower grade astrocytoma. Each subtype is characterized by distinct genetic events (Ohgaki and Kleihues 2013). Men are usually at a higher risk than women for being diagnosed with GBM, and the peak incidence is at 60–75 years of age for both sexes (Yang et al. 2019). Despite recent advances in therapy, the outlook for GBM remains poor with a median post-diagnosis patient survival of fewer than 15 months (Grossman and Batara 2004). Due to the high rate of genetic mutations in GBM, it has become necessary to target several pathways simultaneously for more effective therapies. Different signalling pathways are aberrantly activated in gliomagenesis including Janus kinase (JAK)/ signal transducers and activators of transcription (STAT) (Atkinson et al. 2010) and Notch pathways (Purow et al. 2005).

STAT is one of the best known deregulated pathways in GBM. As transcription factors, STAT proteins are involved in multiple intracellular functions, including cell proliferation, differentiation, survival, angiogenesis, and immune responses (Wang et al. 2020). Constitutive JAK/STAT activation is also associated with GBM tumourigenesis for inhibiting apoptosis while promoting cell growth and invasion (Al Zaid Siddiquee and Turkson 2008; Latha et al. 2013; Cao et al. 2011). Therefore, targeting STATs is thought of as a valuable strategy in cancer therapy including for GBM, and several STAT inhibitors have been developed as potential anticancer agents (Nelson et al. 2011; Kim et al. 2020).

Another aberrantly activated pathway is the Notch pathway in GBM; Notch is involved in the maintenance of neural stem cells and regulating cell fate decision in both the developing brain and the adult brain (Pierfelice et al. 2011). This pathway is evolutionarily conserved in mammals, having four receptors (Notch1–4), five ligands (Jag1/2, Dll1/3/4), and their downstream machinery (Previs et al. 2015). After binding with their ligands, Notch receptors are activated by a series of cleavage events involving ADAM metalloproteases in complex with γ-secretase, releasing the Notch receptor intracellular domain (NICD), that in turn regulates target genes such as Hes and Hey (Yin et al. 2010). In human GBM, the Notch ligand, Jagged1, is most abundantly expressed (Purow et al. 2005). Activated Notch receptors can act as oncogenes (Weng et al. 2004) or tumour suppressors (Hanlon et al. 2010) to modulate tumourigenesis, depending on their cellular context (Siebel and Lendahl 2017). Different interactions of the Notch pathway with other pathways have also been documented (Dotto 2009). Notch signalling by promoting epithelial to mesenchymal transition (EMT) and angiogenesis plays a key role in the maintenance and metastasis of tumours (Leong et al. 2007). Notch signals also confer resistance to radiation and chemotherapeutic agents (Meng et al. 2009). Although Notch has been found in regulating multiple steps in tumourigenesis, the exact role of Notch in glioma development is still unclear. Due to extensive heterogeneity in GBM, the role of Notch is also not consistent, and the divergence of Notch expression in GBM caught our attention. It was also thought that exploring the prevalent crosstalk of the Notch pathway with other pathways could prove useful in developing effective combinatorial cancer therapies (Ranganathan et al. 2011).

In our study, we observed that STAT inhibitors significantly activated Notch signalling in glioblastoma cells. Therefore, we also targeted Notch signalling alongside targeting STATs and studied the effects of this combination in GBM cells. The small molecule inhibitors, pimozide (PMZ) as STAT5 inhibitor, S3I-201 for inhibiting STAT3 phosphorylation in combination with γ-secretase inhibitor (DAPT), were studied for blocking STAT signalling and Notch activation in our system. Our results suggest that dual inhibition of STAT and Notch pathways strongly impairs viability and tumourigenesis in GBM cells. Our findings point to the potential of simultaneous targeting of these two pathways in GBM treatment.

## Materials and methods

### Patient datasets

Correlation analysis for gene expression in GBM cases was performed using the microarray TCGA search engine for the dataset of 530 GBM patient samples in The Cancer Genome Atlas Program site (TCGA, 2008; http://tcga-data.nci.nih.gov).

### Cell culture

Human glioblastoma cell lines (LN18, A172, LN229, and U87MG) and the astrocyte cell line were purchased from the American Type Culture Collection (ATCC). The cells were maintained in high-glucose Dulbecco’s Modified Eagle Medium (DMEM) (Biowest), supplemented with 10% fetal bovine serum (Biowest) and 1% penicillin/streptomycin (Lonza) and in a 37°C, 5% CO_2_ atmosphere incubator.

### Drugs and treatments

S3I-201 (Cayman Chemical Company), pimozide (PMZ) (Sigma–Aldrich, USA) and DAPT (Sigma–Aldrich, US) were dissolved in DMSO and stocks were stored at −20°C. Cells were treated with S3I-201 (150 or 300 µM) or PMZ (15 or 20 µM) and in combination with DAPT (10 or 20 µM). Cells were harvested or analyzed at 24 h post-treatment.

### Western blot analysis

Treated whole cells were harvested and lysed in RIPA lysis buffer (50 mM Tris-HCl, 150 mM NaCl, 1% NP-40, 1% sodium deoxycholate, 0.1% SDS) supplemented with 1 mM NaF, 1 mM Na_3_VO_4_ and a protease inhibitor cocktail (Sigma–Aldrich, USA). Total protein content was quantified using a BCA protein assay kit (Thermo Fisher Scientific, USA) following the manufacturer’s instructions. Total protein at 25∼30 μg for each sample was separated by SDS–PAGE; the proteins were then transferred onto PVDF or nitrocellulose membranes. The membranes were blocked in 5% skimmed milk for 1 h and at room temperature (RT) and were then incubated with primary antibodies overnight at 4°C. Anti-Jagged1 and anti-phospho-Stat5a/b (Tyr694/699) antibodies were purchased from Santa Cruz Biotechnology. Anti-cleaved Notch1 (Val1744), anti-phospho-Stat3 (Tyr705), anti-cleaved PARP (Asp214), anti-cleaved caspase 3 (Asp175) and anti-GAPDH antibodies were obtained from Cell Signalling Technology. Following the incubation with the primary antibodies, the membranes were washed and probed with HRP-conjugated secondary antibodies 1 h at RT. The bound antibodies were detected with a chemiluminescent reagent (Millipore, USA) using an enhanced chemiluminescence imaging system (Fusion FX, Vilber Lourmat, France).

### RNA extraction and RT-qPCR

Total RNA was extracted from treated cells using the TRIzol reagent (Invitrogen, USA). cDNAs were then synthesized from 2 µg of total RNA using the MLV-reverse transcriptase (Enzynomics, Korea) according to the manufacturer’s protocol. RT-qPCR was performed using a TOPreal qPCR 2X PreMIX SYBR Green with high ROX (Enzynomics, Korea) with primers specific to target genes in the StepOnePlus Real-Time PCR system (Applied Biosystems, USA). The primers specific to target genes are listed in Supplementary Table 1. The RT-qPCR conditions were: initial denaturation at 95°C for 5 min, followed by 50 cycles of 95°C for 10 sec, 60°C for 20 sec, and 72°C for 10 sec. Expression levels of target mRNAs were obtained using the ddCt method and were normalized to the GAPDH gene. The fold changes calculated were relative to the control group.

### Cell viability assay

Cell viability was evaluated by the CCK-8 assay (Dojindo, Japan). Briefly, 1 × 10^5^ cells per well were plated in 96-well plates; after 24 h, the cells were treated with PMZ (10–30 µM), S3I-201 (75–300 µM), DAPT (10–20 µM) or in combinations of PMZ + DAPT, S3I-201+DAPT for 24 h at 37°C. CCK-8 solution at 10 µl was added to each well and incubated for two to four h at 37°C. Absorbance was measured at 450 nm with a spectrophotometer (SpectraMax Plus 384, Molecular Devices, USA).

### Cell migration assay

LN18 and A172 cells were seeded in a 6-well plate and allowed to reach 90% confluence. At that point scratches at their monolayer were made using 1 ml pipet tips. The cells were further incubated for 24 h in growth media in presence of various treatments. Photographs were taken of the scratched/wounded area at 0 and 24 h with a digital camera. The extent of cell migration was calculated according to the following formula:

$$\begin{array}{rl} & Migration\;Index\\ & =\frac{Width\;of\;the\;wound_{0\;h}-Width\;of\;the\;wound_{24\;h}}{Width\;of\;the\;wound_{0\;h}}\\ & \;\times 100 \end{array}$$

### Cell invasion assay

The invasion assay was performed using a specialized invasion chamber (Corning BioCoat Matrigel Invasion Chamber, USA). Briefly, a total of 5 × 10^6^ cells/2 ml were plated in the upper chamber in serum-free media (100 µl). The cells were treated simultaneously with respective treatments and their control. The upper chambers were placed in a 24-well plate containing media with 10% FBS. After 24 h incubation at 37°C, 5% CO^2^ atmosphere, the cells were washed with PBS. The invading cells were fixed in 3.7% formaldehyde, permeabilized with 100% methanol and stained with 0.4% crystal violet. The non-invading cells on the upper surface of the membrane were gently removed with a cotton swab. Photographs were taken under the light microscope with a digital camera. The calculation of the invading cells was according to the following formula:

$$Invasion\;Index=\frac{\text{\%}\;Invasion\;Test\;Cell}{\text{\%}\;Invasion\;Control\;Cell}$$

### Statistical analysis

GraphPad Prism was used for all statistical analyses (version 5.0; GraphPad Software, USA). Data are presented as mean ± SEM. All experiments were performed at least in triplicate. Data were analyzed by two-tailed Student’s unpaired *t*-test. *P* < 0.05 (*), *P* < 0.01 (**), and *P* < 0.001 (***) were considered statistically significant. Correlations for gene expression were with Pearson’s product-moment correlation coefficient (*ρ*).

## Results

### STAT3, STAT5A and STAT5B expression correlate with jagged1 expression in GBM patients

**Figure 1.**
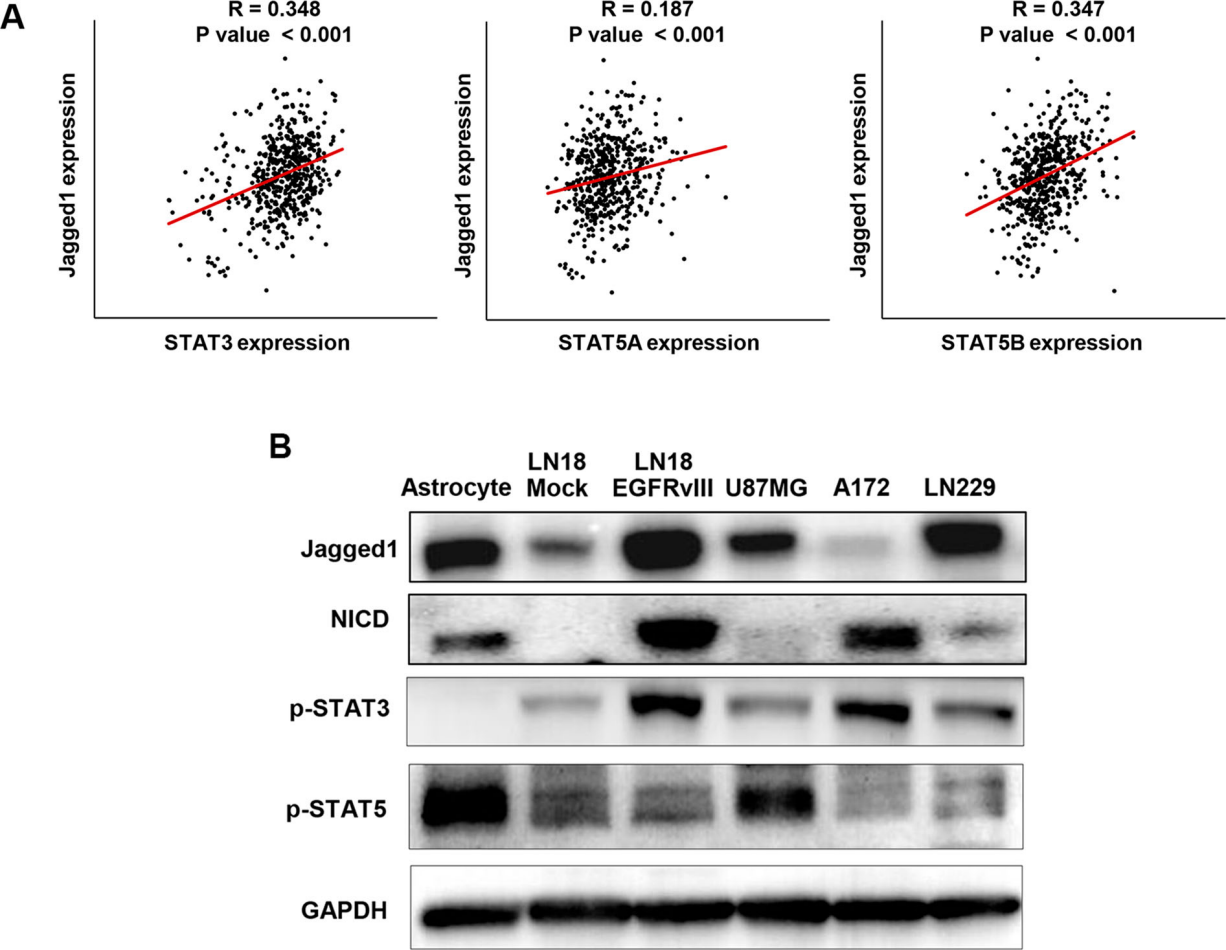
Activation of Jagged/Notch and STATs in different glioblastoma cell lines and expression correlation of different STATs with Jagged1 in glioblastoma patients. (A) Expression correlation of STAT3, STAT5A and STAT5B with Jagged1 was performed using a TCGA dataset pool of 530 GBM patients. STAT3 (*R *=* *0.348, *P *<* *0.001), STAT5A (*R *=* *0.187, *P *< 0.001) and STAT5B (*R *=* *0.347, *P *< 0.001) expression levels significantly correlated with those of Jagged1 in human glioblastoma patients. (B) Western blot analysis of Jagged1, NICD, p-STAT3 and p-STAT5 protein levels in a panel of human glioblastoma cell lines. An astrocyte cell line was used as a normal cell control and GAPDH was used as the loading control.

STAT and Notch pathways are aberrantly expressed in GBM as previously reported. We, therefore, initially sought to see whether *STAT3*, *STAT5A*, and *STAT5B* expression levels correlated with that of Notch ligand-*Jagged1* in human GBM patients using the web-based bioinformatics database engine of TCGA at the National Cancer Institute. From that analysis, although *STAT5A* expression levels correlated slightly with *Jagged1* levels, a marked correlation was observed between *STAT3* and *Jagged1*, and also between *STAT5B* and *Jagged1* (Figure 1(A))*.* To further confirm the co-activation of these pathways in GBM samples, we examined a panel of human GBM cell lines compared with normal astrocyte cells to analyze the basal expression level of Jagged1, Notch receptor intracellular domain (NICD), p-STAT3 and p-STAT5. These were found to be activated differentially among the cell lines (Figure 1(B)) and suggested crosstalk between STAT and Notch signalling in GBM.

### STAT inhibitors induce Notch signalling in glioblastoma cells

**Figure 2.**
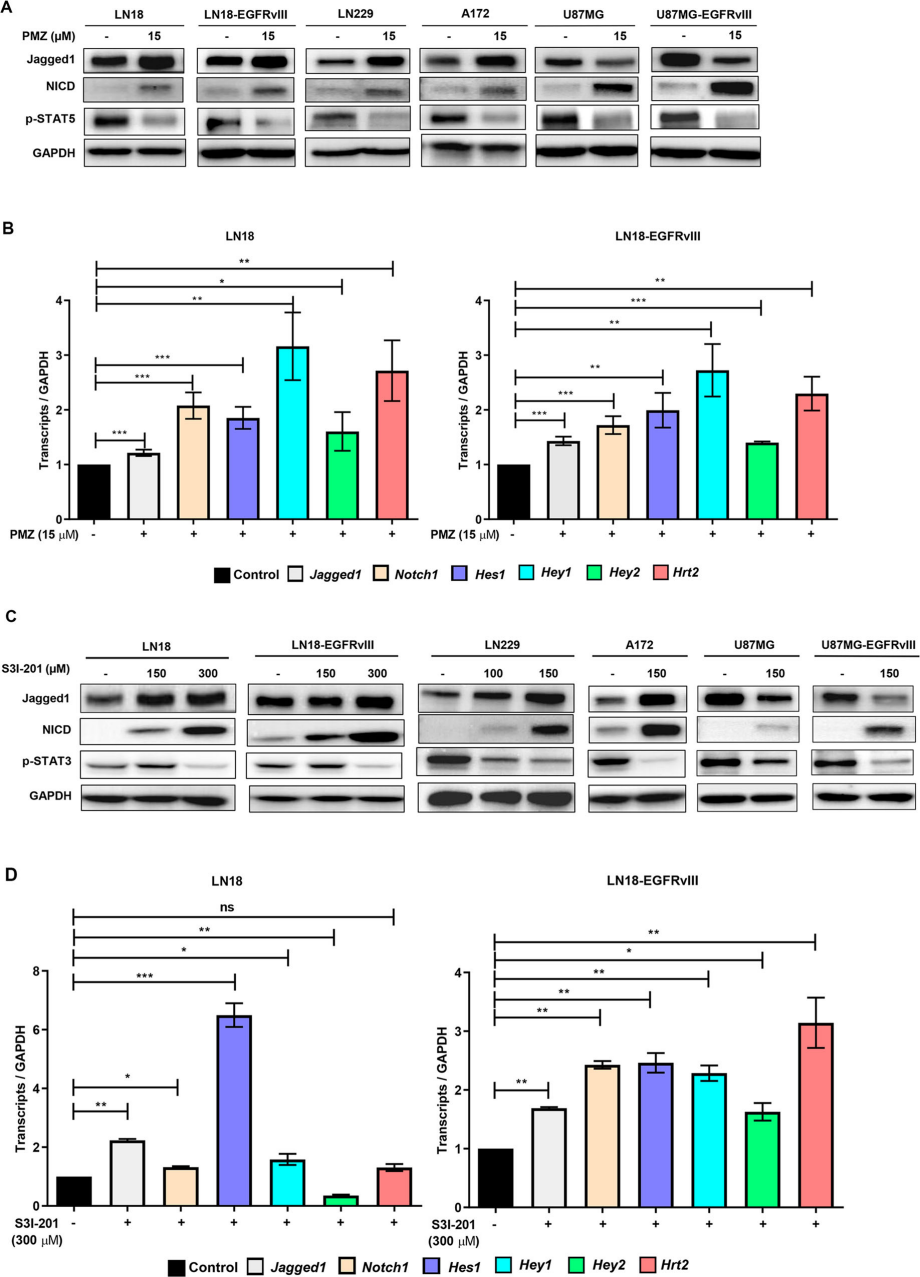
STAT inhibitors induce Notch signalling in glioblastoma cells. A panel of glioblastoma cell lines (LN18, LN18-EGFRvIII, LN229, A172, U87MG-EGFRvIII) were checked for their Jagged1 and NICDs levels by Western blotting 24 h post-treatment in (A) with PMZ (15 µM) and (C) with S3I-201 (100–300 µM). Vehicle only (DMSO) was used as control. GAPDH served as a loading control. Relative cellular mRNA levels for Jagged1, Notch1 and Notch target genes (Hes1, Hey1, Hey2, Hrt2) were quantified by RT-qPCR in LN18 and LN18-EGFRvIII cells treated in (B) with PMZ (15 µM) and (D) with S3I-201 (300 µM) 24 h post-treatment. DMSO only treatment was used as control, with relative expression defined as 1.0. Individual samples in the graphical data are shown with mean ± SEM. *P < 0.05, **P < 0.01, ***P < 0.001, using the Student’s unpaired *t*-test.

To further verify the crosstalk between STAT and Notch pathways in GBM, we next checked Notch regulation by blocking STATs in glioblastoma cells. We used the STAT5 inhibitor PMZ (15 µM) and STAT3 inhibitor S3I-201 (100–300 µM) for a 24 h treatment of LN18, LN18-EGFRvIII, LN229, A172, U87MG, and U87MG-EGFRvIII cells and checked for changes in their Jagged1 and NICD protein expression levels (Figure 2(A, C), respectively). PMZ treatment blocked STAT5 phosphorylation and also significantly upregulated Jagged1 expression in all cells except U87MG and U87MG-EGFRvIII where Jagged1 expression was appreciably downregulated (Figure 2(A)). S3I-201 treatment also upregulated Jagged1 expression in all the cell lines except U87MG and U87MG-EGFRvIII cells where Jagged1 expression was significantly decreased (Figure 2(C)). However, for all these cell lines, NICD expression was significantly elevated by both PMZ and S3I-201 treatments (Figure 2(A, C), respectively). These results indicate a possible negative regulation of Notch signalling by STATs in GBM cells. For further confirmation, we also examined expression changes for *Jagged1* and *Notch1* and the panel of Notch target genes, *Hes1, Hey1, Hey2,* and *Hrt2*, by RT-qPCR following PMZ and S3I-201 treatment of LN18 cells (Figure 2(B, D), respectively) and also for other glioblastoma cells (Figure S1). Treatment of LN18 and LN18-EGFRvIII cells with 15 µM PMZ showed a significant upregulation of *Jagged1, Notch1* and Notch target genes, *Hes1, Hey1, Hey2,* and *Hrt2* (Figure 2(B)). In addition, treatment of LN18 cells with 300 µM S3I-201 also upregulated the expression of *Jagged1* and *Notch1*; among the Notch target genes, only *Hes1* was significantly upregulated. For LN18-EGFRvIII cells, *Jagged1, Notch1* and including all the Notch target genes showed significantly elevated expression (Figure 2(D)). Differentially activated *Jagged1, Notch1*, and Notch target genes expression in other GBM cells with either PMZ or S3I-201 treatment are shown in Figure S1(A, B, C, and D). In summary, we observed increased levels of Notch signalling shown at both RNA and protein levels for pathway markers seen by treatment with STAT inhibitors in glioblastoma cells. These results support the notion that STATs negatively regulate Notch signalling in GBM cells.

### STAT inhibitors induce apoptosis in GBM cells and a combination of STAT and Notch inhibitors is more effective in increasing apoptosis in treated cells

**Figure 3.**
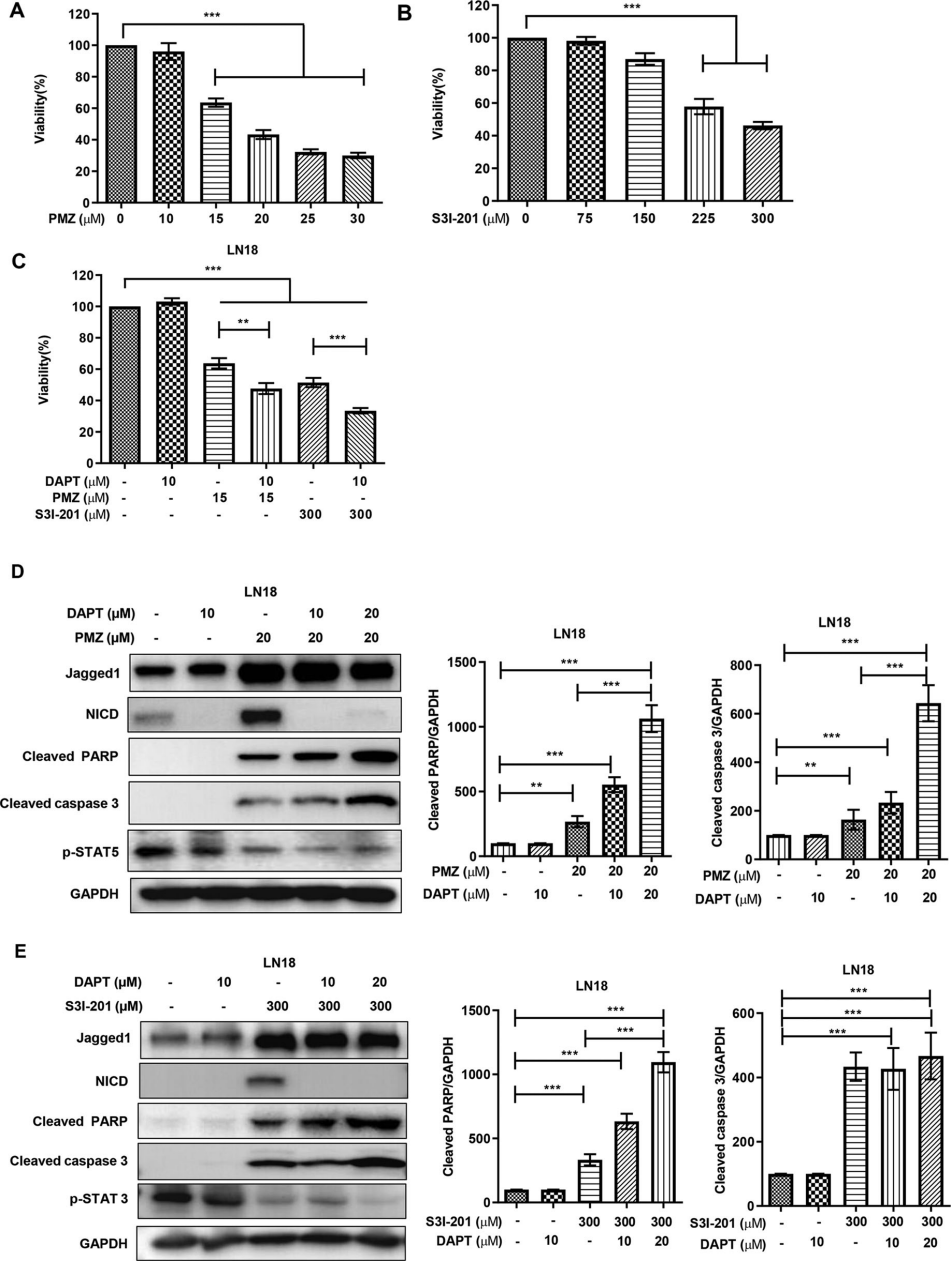
STAT inhibitors reduce GBM cell proliferation while inducing apoptosis and a combination of STAT and Notch inhibitors further increase the apoptosis levels. LN18 cells were treated with (A) PMZ (10–30 µM), (B) S3I-201 (75–300 µM) for 24 h for cell viability changes quantitated by the CCK8 assay. In (C), DAPT (10 µM), PMZ (20 µM), S3I-201 (300 µM) alone and a combination of PMZ + DAPT or S3I-201+DAPT were used to treat LN18 cells for cell viability changes measured by the CCK8 assay. LN18 cells were treated with (D) DAPT (10 µM), PMZ (20 µM) and combination of PMZ + DAPT (20 + 10, 20 µM), and in a separate experiment, the cells were treated with (E) DAPT (10 µM), S3I-201 (300 µM) and combination of S3I-201+DAPT (300 + 10, 20 µM) for 24 h. Cleaved PARP and cleaved caspase 3 levels were checked by Western blotting. DMSO only treatment was used as control and was assigned a value of 100 in bar graphs for densitometric analysis of the blots. Individual samples are shown with mean ± SEM. *P < 0.05, **P < 0.01, ***P < 0.001, using the Student’s unpaired *t*-test.

For cytotoxicity changes due to STAT inhibitors in GBM cells, we used the CCK-8 cell viability assay with dose optimization for PMZ (10–30 µM) and S3I-201 (75–300 µM) in LN18 cells, read at 24 h post-treatment. Viability in the cells was reduced in a dose dependent manner with 20 µM PMZ and 300 µM S3I-201 giving a 50% viability drop in LN18 cells (Figure 3(A, B), respectively). Since STAT inhibitors were showing significant Notch activation (Figure 2), we next combined Notch inhibition by the γ-secretase inhibitor DAPT with the STAT inhibitors. Combinations of PMZ + DAPT and S3I-201+DAPT were tested for viability changes in LN18 and A172 cell lines. PMZ + DAPT or S3I-201+DAPT combinations showed stronger inhibitory effects than PMZ (20 µM) or S3I-201 (300 µM) alone; DAPT (10 µM) treatment alone did not significantly affect the viability of LN18 and A172 cells (Figure 3(C) and S2A, respectively). These data indicate that this dual mode of inhibition is highly effective in reducing GBM cell viability. To determine whether the cell viability reductions were due to induction of apoptosis, we further checked the status of cellular apoptosis markers PARP and caspase 3 for cleavage in LN18 and A172 cells post-treatment with PMZ alone, PMZ + DAPT, S3I-201 alone, and S3I-201+DAPT. Elevated levels of cleaved PARP and cleaved caspase 3 were detected after combination treatment in both LN18 (Figure 3(D, E), respectively) and A172 (Figure S2(B and C), respectively) cells. DAPT alone did not lead to apoptotic marker changes. Taken together, these results suggest that STAT inhibitors PMZ or S3I-201 treatment induce apoptotic cell death more effectively in a combination with DAPT.

### Combined inhibition of STAT and Notch signalling reduced migration and invasion in glioblastoma cells

**Figure 4.**
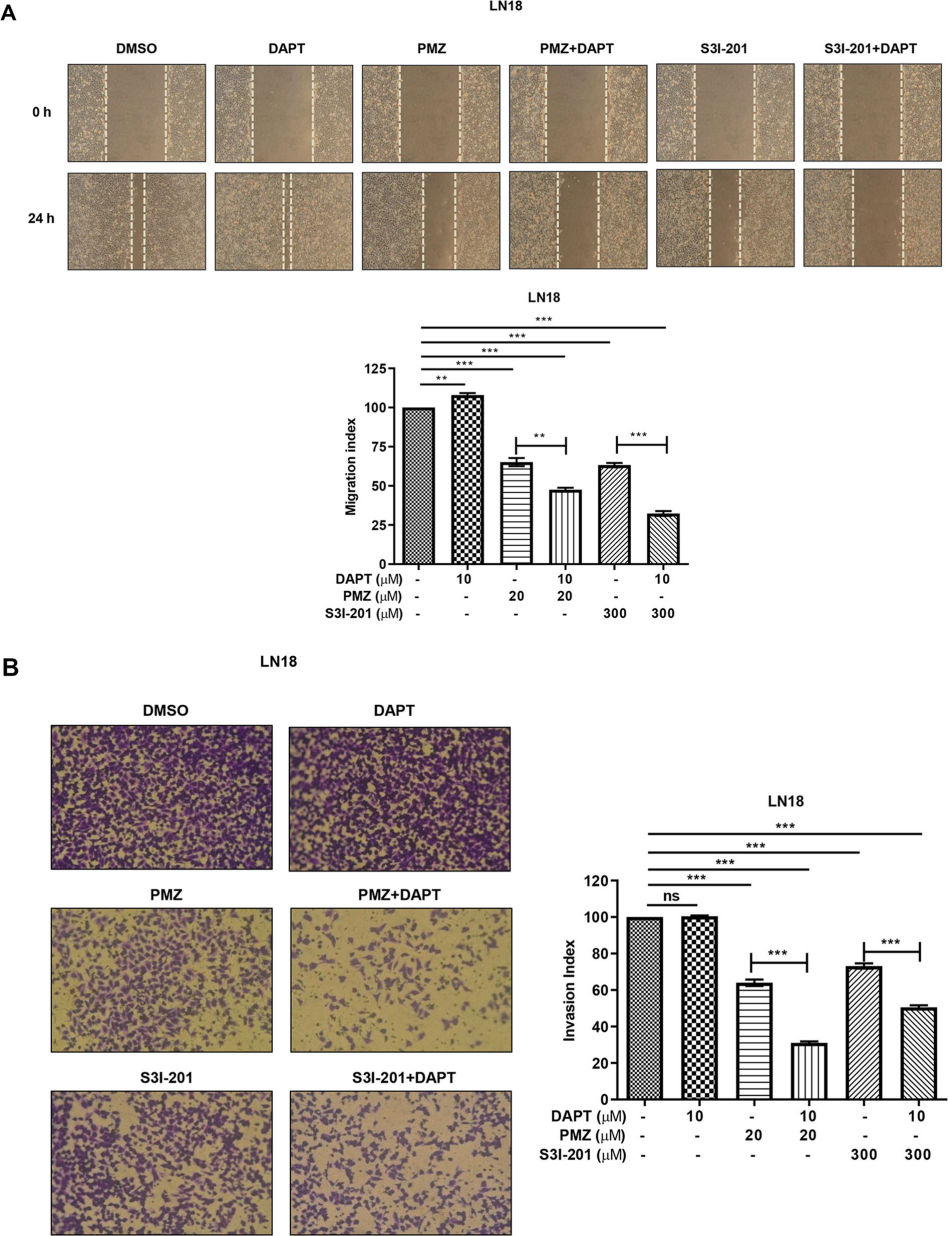
Combined inhibition of STAT and Notch pathways blocks migration and invasion in GBM cells. (A) Wound healing experiments for LN18 cells treated with DAPT (20 µM), PMZ (20 µM), S3I-201 (300 µM) or a combination of PMZ + DAPT and S3I-201+DAPT. Dotted lines represent the edge of the wound. The migration index was calculated as described in the Methods section and plotted as bar graphs. (B) LN18 cells were treated with DAPT, PMZ, S3I-201 or a combination of PMZ + DAPT and S3I-201+DAPT for 24 h; the same doses mentioned for the migration assay were also used in the invasion assay. The invasion index was calculated as described in the Methods section and plotted as bar graphs. DMSO only treatment was used as control and was assigned a value of 100 in the plots. Individual samples are shown with mean ± SEM. *P < 0.05, **P < 0.01, ***P < 0.001, using the Student’s unpaired *t*-test.

In glioblastoma, extra-cranial metastases are rare; however, migration and invasion of the cancer cells are significant features. For DAPT, PMZ, S3I-201 treatments given alone and the combination PMZ + DAPT or S3I-201+DAPT treatments, we conducted wound healing assays on glioblastoma cells. The assays reflect migration potential changes in the study cells. Cell migration was blunted with either PMZ (20 µM) or S3I-201 (300 µM) treatment compared to control cells; in the presence of DAPT (20 µM), wounding migration levels were most affected in combination with either PMZ or S3I-201 in LN18 and A172 cells (Figure 4(A) and S3, respectively). DAPT alone slightly increased migration in LN18 cells (Figure 4(A)) and no obvious change in the case of A172 cells (Figure S3). LN18 cells were then used to determine the effect of PMZ or S3I-201 and their combinations with DAPT on the invasiveness of glioblastoma cells using Matrigel-coated transwell chambers. Cells were treated with similar concentrations and combinations of inhibitors as mentioned previously for the wound healing assay. PMZ (20 µM) and S3I-201 (300 µM) treatment reduced the Matrigel invasion of LN18 cells and combination treatments PMZ + DAPT or S3I-201+DAPT displayed a higher impact in reducing invasiveness than PMZ or S3I-201 alone. Treatment with DAPT (20 µM) alone did not have any reduced invasion effects (Figure 4(B)). These results show that combined treatments of STAT and Notch inhibitors more effectively inhibit migration and invasion of glioblastoma cells than either single treatment.

## Discussion

Glioblastoma is a highly aggressive type of primary brain tumour, well known for its diffuse invasion, heterogeneity, resistance to treatment and poor patient survival (Kim et al. 2020a; Wen and Kesari 2008). In recent years, research has uncovered several potential targets which may lead to improved patient outcomes. The wide-ranging heterogeneity and high metastatic nature of GBM suggest that targeting a single tumour signalling pathway may not be promising enough in reducing tumour aggressiveness and improving patient survival. Targeting several signalling pathways with overlapping roles may obviate drug resistance and increase the probability of treatment response. In addition, certain compensatory mechanisms in one pathway may be blocked if multiple pathways are targeted (Palagani et al. 2014).

Various studies have indicated a role for STAT and Notch signalling in the progression of glioblastoma (Wang et al. 2018). Both pathways are involved in various cellular activities, including differentiation, proliferation, apoptosis, and cell survival. Although individually blocking these two pathways has yielded some benefit, a combination treatment has not been yet investigated (Mukthavaram et al. 2015; Xu et al. 2016). To our knowledge, crosstalk between these two pathways has not been explored in glioblastoma, although in breast cancer cells, Notch signalling promotes IL-6/JAK/STAT and is regulated by p53/IKKα/IKKβ (Jin et al. 2013). However, crosstalk between STAT and Notch signalling has been noted in glial cells (Kamakura et al. 2004), where Notch effectors Hes1 and Hes5 associate with JAK2 and STAT3, and promote the formation of the complex between JAK2 and STAT3, leading to STAT3 phosphorylation/activation. Notch signalling has also been found to mediate radioresistance in glioma stem cells (Wang et al. 2010). Thus, we sought to investigate targeting these two prominent tumour signalling pathways in our study system.

We confirmed that several glioblastoma cell lines have activated STAT and Notch markers (Figure 1(B)). By targeting STATs with different small molecule inhibitors, we also observed that these inhibitors also increased Notch signalling (Figure 2). As STAT inhibitors showed increased apoptotic cell death in glioblastoma cell lines (Figure 3 and S2), we hypothesized that combination treatment with DAPT Notch pathway inhibitor could be a better strategy in reducing proliferation and migration in these cells. We found that combination treatments led to significant decreases in cell proliferation and promoted apoptotic cell death (Figure 3 and S2). Although the STAT inhibitors PMZ and S3I-201 singly could induce significant cell death, DAPT alone did not affect; however, treatment with PMZ or S3I-201 in combination with DAPT saw significantly augmented increases in cell apoptosis. The combination treatments of PMZ + DAPT or S3I-201 + DAPT also showed significantly higher levels of inhibition in cell migration and invasion in the glioblastoma cells (Figure 4(A, B)). We speculate that Notch activation induced by STAT inhibitors treatment is involved in drug resistance, activating certain pro-survival pathways. Therefore, inhibition of Notch signalling sensitizes glioblastoma cells to drugs such as PMZ and S3I-201, accounting for significant gains in inhibition in the study parameters. Certainly, our findings support crosstalk between Notch and STAT signalling. Based on other publications, a robust inhibition in tumour progression is seen by the fact that both STATs and Notch are involved in GBM development and progression (Bazzoni and Bentivegna 2019). Single pathway blocking is not as effective on GBM proliferation and tumourigenesis as combinational treatments and our data suggest that the antitumour effect of PMZ or S3I-201 can be improved by concurrent inhibition of Notch signalling. Although single pathway targeting shows some antitumour activity, the dual pathway targeting gains in efficacy are noteworthy.

We have to mention that our study is limited in defining the details of the underlying mechanism for Notch activation by STAT inhibitors and identifying the links between the two pathways. Furthermore, the biological role of Notch activation by STAT inhibitors is still not known. Whether increased activation of Notch signalling from inhibition of JAK/STAT is specific and direct or whether it is an indirect response to the added stress to the cells is currently not known and would require additional experiments. It will also be essential to identify any additional biological markers for patient populations that may benefit most from such a treatment regimen. Further studies will be required to dissect the roles of Notch and STAT signalling crosstalk. It already seems quite promising to target these two pathways with the additional possibility of further combining them with classical chemotherapeutic or radiation treatment. The safety profile of these combination treatments needs to be determined in preclinical models of glioblastoma. The safety profile of these compounds may also be improved by attaching cancer-targeting moieties to these small molecules. In conclusion, we provide the first evidence for significant gains in the dual treatment of GBM cells with DAPT with S3I-201 or PMZ compared with single-agent treatments in reducing proliferation and migration. Our data provide evidence that simultaneous targeting of Notch and STAT signalling could improve efficacy in the treatment of GBM and support the basis for further studies on therapeutics jointly targeting these two pathways.

## Supplementary Material

Supplemental Material
