# Supplementary material for: Combined inhibition of STAT and Notch signalling effectively suppresses tumourigenesis by inducing apoptosis and inhibiting proliferation, migration and invasion in glioblastoma cells
Source: Anim Cells Syst (Seoul). 2021 Jun 25;25(3):161–70. doi: 10.1080/19768354.2021.1942983 (PMC8253205; doi:10.1080/19768354.2021.1942983)
Supplement: Supplemental Material [file TACS_A_1942983_SM0721.zip › Supplementary Figure Legends.docx]

**Supplementary Figure Legends**

**Supplementary Figure 1.** STAT inhibitors induce Notch signaling in glioblastoma cells. Cellular mRNA expression of Jagged1, Notch1 and Notch target genes (Hes1, Hey1, Hey2, Hrt2) were checked in (A) A172, (B) LN229, (C) U87MG and U87MG-EGFRvIII cells treated with PMZ (15 µM) and for 24 h. Same genes were checked in (D) LN229, (E) U87MG and U87MG-EGFRvIII cells treated with S3I-201 (150 µM) and for 24 h in a separate experiment. DMSO only treatment was used as control and expressed as 1.0 in graphical data. The individual samples are shown with mean ± SEM. *P < 0.05, **P < 0.01, ***P < 0.001, using the Student's unpaired t-test.

**Supplementary Figure 2.** STAT inhibitors reduce GBM cell proliferation while inducing apoptosis and combination of STAT and Notch inhibitors increase apoptosis. (A) PMZ (15 µM), S3I-201 (300 µM), DAPT (10 µM), and combination of PMZ+DAPT or S3I-201+DAPT were used to treat A172 cells to examine cell viability by the CCK8 assay. (B) A172 cells were treated with DAPT (5 µM), PMZ (20 µM) and combination of PMZ+DAPT. (C) In a separate experiment, cells were treated with DAPT (10 µM), S3I-201 (300 µM) and combination of S3I-201+DAPT for 24 h. Cleaved PARP and cleaved caspase 3 levels are shown via Western blotting. DMSO only treatment was used as control and expressed as 100 in bar graphs for densitometric analysis of the Western blots. The individual samples are shown with mean ± SEM. *P < 0.05, **P < 0.01, ***P < 0.001, using the Student's unpaired t-test.

**Supplementary Figure 3.** Combined pharmacological inhibition of STAT and Notch inhibits the migration of glioblastoma cells. Wound healing experiments of A172 cells treated with DAPT (20 µM), PMZ (20 µM), S3I-201 (300 µM) or combination of PMZ+DAPT and S3I-201+DAPT. The dotted lines represent the edge of the wound. The migration index was calculated as described in the methods section and was plotted in bar graphs. DMSO only treatment was used as control and expressed as 100 in graphical data. The individual samples are shown with mean ± SEM. *P < 0.05, **P < 0.01, ***P < 0.001, using the Student's unpaired t-test.
