# Supplementary material for: Combined inhibition of STAT and Notch signalling effectively suppresses tumourigenesis by inducing apoptosis and inhibiting proliferation, migration and invasion in glioblastoma cells
Source: Anim Cells Syst (Seoul). 2021 Jun 25;25(3):161–70. doi: 10.1080/19768354.2021.1942983 (PMC8253205; doi:10.1080/19768354.2021.1942983)
Supplement: Supplemental Material [file TACS_A_1942983_SM0721.zip › Supplementary Table 1.docx]

**Supplementary** **Table1. Primers used for RT-qPCR amplification**

| **Primer** | **Direction** | **Sequence** |
| --- | --- | --- |
| Jagged1 | Forward | 5’-ACACGGTCCCCATTAAGGA-3’ |
|  | Reverse | 5’-GTCCAGTTCGGGTGTTTTGT-3’ |
| Notch1 | Forward | 5’-TTGTAGGAGACTTGCCAGAG-3’ |
|  | Reverse | 5’-GCATGACACACAACAGACTC-3’ |
| Hes1 | Forward | 5’-GGCCAGTTTGCTTTCCTCAT-3’ |
|  | Reverse | 5’-TTTAGAGTCCGGAGGGAAGA-3’ |
| Hey1 | Forward | 5’-CTCGCACACCATGATCACTT-3’ |
|  | Reverse | 5’-AACTGTTGGTGGCCCTGAAT-3’ |
| Hey2 | Forward | 5’-TTGAAGATGCTTCAGGCAACAGGG-3’ |
|  | Reverse | 5’-TCAGGTACCGCGCAACTTCTGTTA-3’ |
| Hrt2 | Forward | 5’-CCAGAAAAAGACGGAGAGGA-3’ |
|  | Reverse | 5’-GCGCGTCAAAGTAACCTTTC-3’ |
| GAPDH | Forward | 5'-AGAAGGCTGGGGCTCATTTG-3' |
|  | Reverse | 5'-AGGGGCCATCCACAGTCTTC-3' |
