# Supplementary figures and images for: Combined inhibition of STAT and Notch signalling effectively suppresses tumourigenesis by inducing apoptosis and inhibiting proliferation, migration and invasion in glioblastoma cells
Source: Anim Cells Syst (Seoul). 2021 Jun 25;25(3):161–70. doi: 10.1080/19768354.2021.1942983 (PMC8253205; doi:10.1080/19768354.2021.1942983)

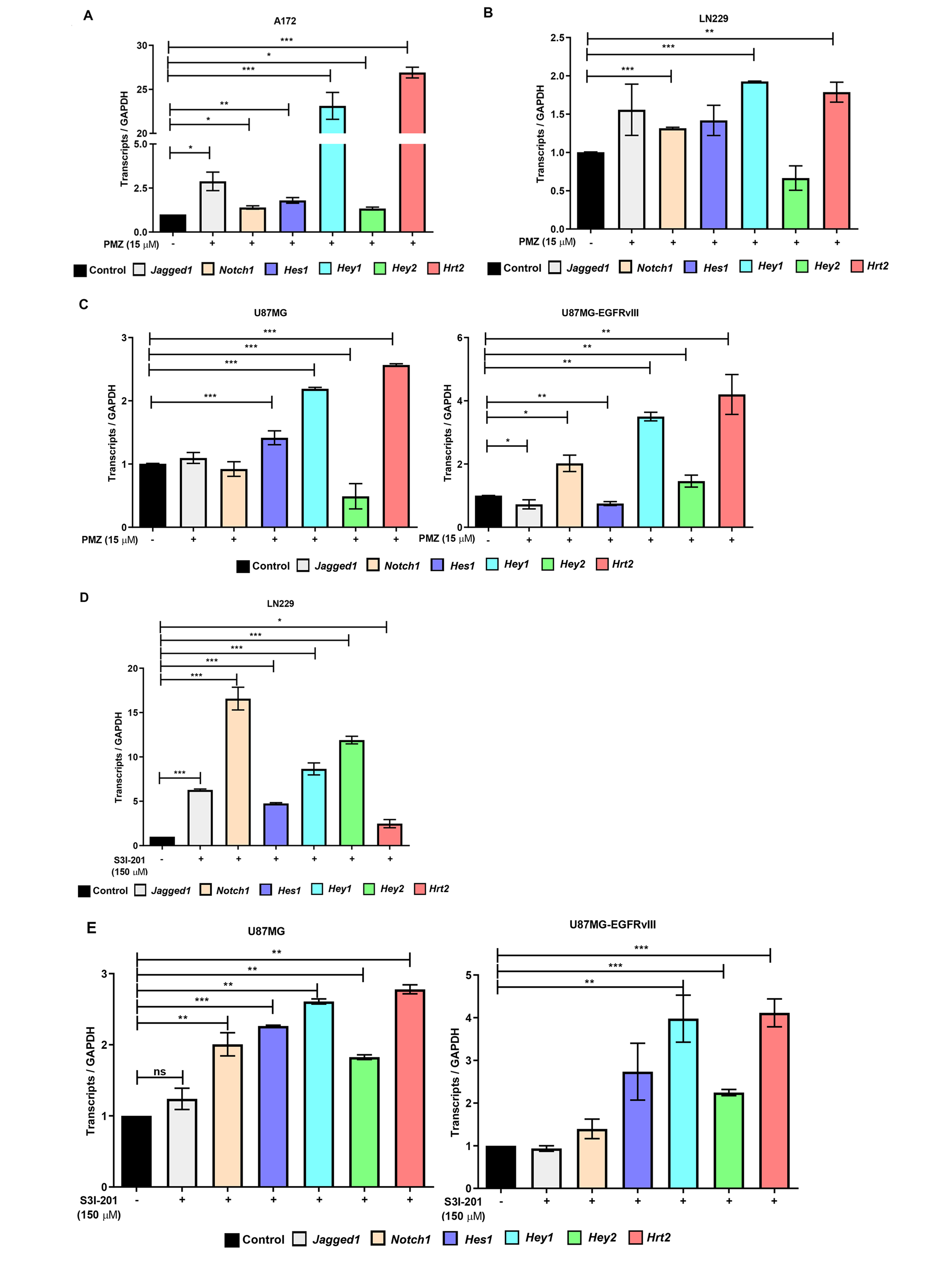

Supplement: Supplemental Material [file TACS_A_1942983_SM0721.zip › Supplementary_ Figure 1.tif]

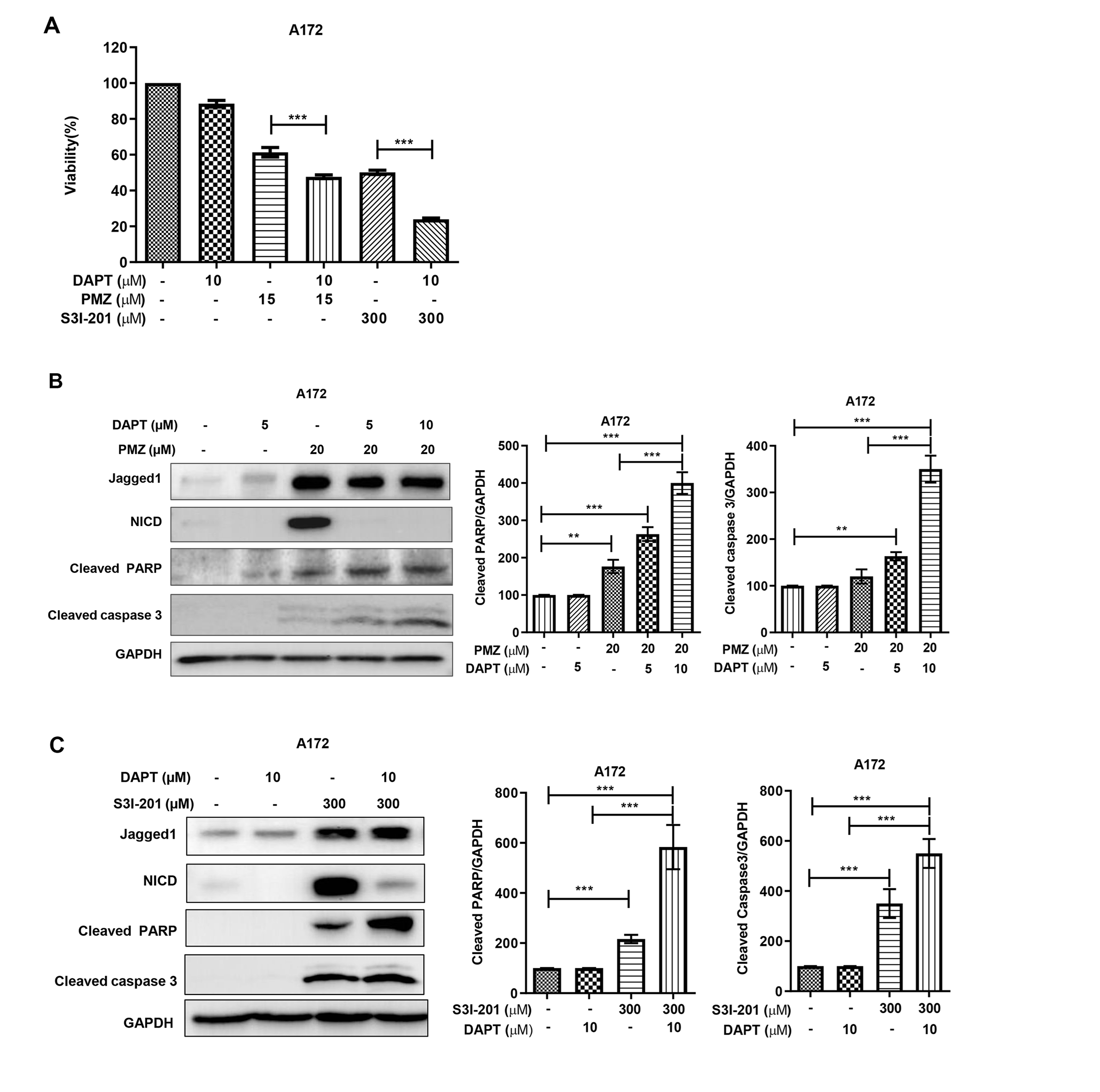

Supplement: Supplemental Material [file TACS_A_1942983_SM0721.zip › Supplementary_ Figure 2.tif]

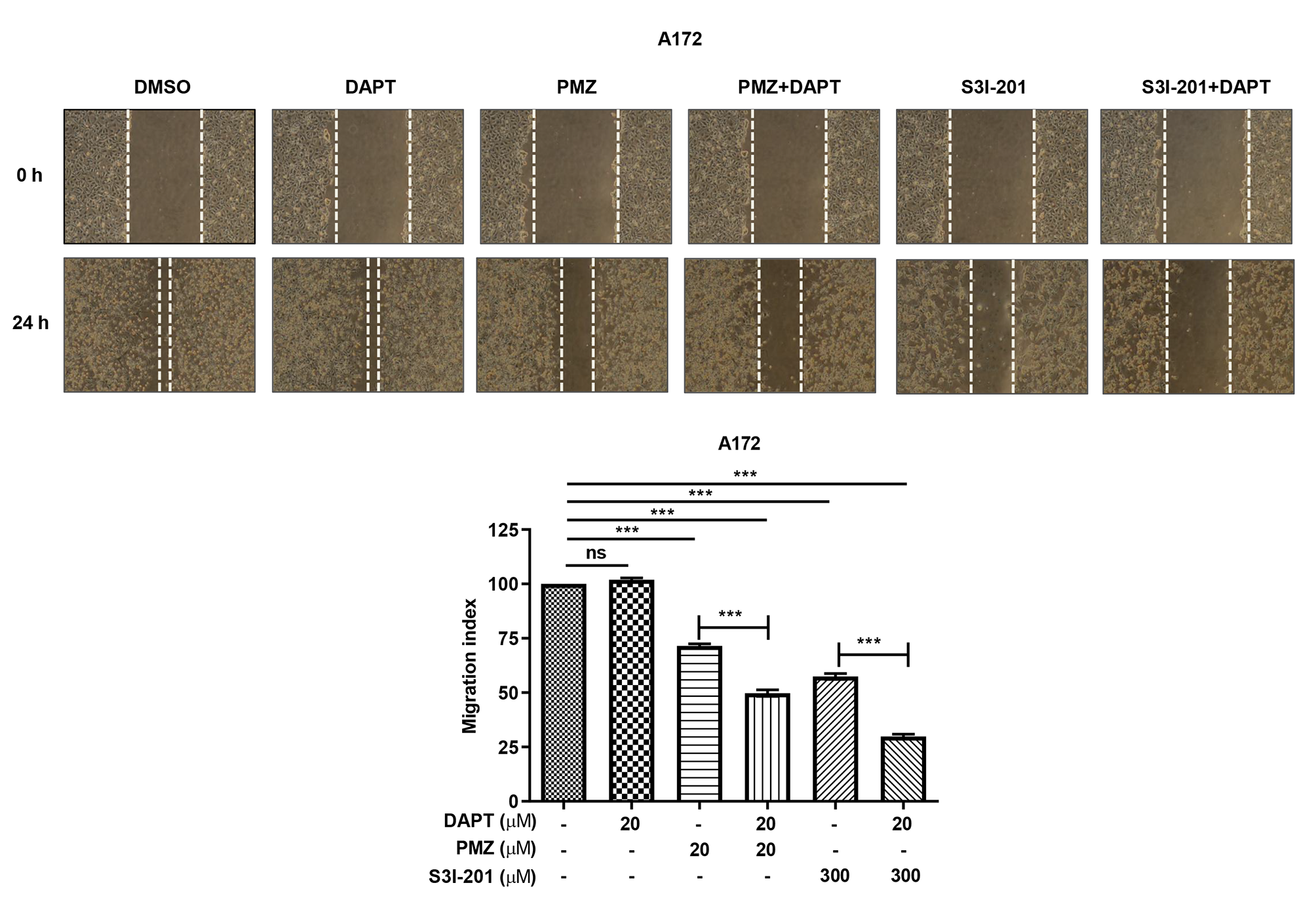

Supplement: Supplemental Material [file TACS_A_1942983_SM0721.zip › Supplementary_ Figure 3.tif]
